# Scalable and interpretable alternative to chart review for phenotype evaluation using standardized structured data from electronic health records

**DOI:** 10.1093/jamia/ocad202

**Published:** 2023-10-17

**Authors:** Anna Ostropolets, George Hripcsak, Syed A Husain, Lauren R Richter, Matthew Spotnitz, Ahmed Elhussein, Patrick B Ryan

**Affiliations:** Department of Biomedical Informatics, Columbia University Irving Medical Center, New York, NY 10032, United States; Department of Biomedical Informatics, Columbia University Irving Medical Center, New York, NY 10032, United States; Medical Informatics Services, New York-Presbyterian Hospital, New York, NY 10032, United States; Division of Nephrology, Department of Medicine, Vagelos College of Physicians and Surgeons, Columbia University Irving Medical Center, New York, NY 10032, United States; Department of Biomedical Informatics, Columbia University Irving Medical Center, New York, NY 10032, United States; Department of Biomedical Informatics, Columbia University Irving Medical Center, New York, NY 10032, United States; Department of Biomedical Informatics, Columbia University Irving Medical Center, New York, NY 10032, United States; Department of Biomedical Informatics, Columbia University Irving Medical Center, New York, NY 10032, United States; Observational Health Data Analytics, Janssen Research and Development, Titusville, NJ 08560, United States

**Keywords:** chart review, phenotyping, observational studies, case adjudication, case ascertainment

## Abstract

**Objectives:**

Chart review as the current gold standard for phenotype evaluation cannot support observational research on electronic health records and claims data sources at scale. We aimed to evaluate the ability of structured data to support efficient and interpretable phenotype evaluation as an alternative to chart review.

**Materials and Methods:**

We developed Knowledge-Enhanced Electronic Profile Review (KEEPER) as a phenotype evaluation tool that extracts patient’s structured data elements relevant to a phenotype and presents them in a standardized fashion following clinical reasoning principles. We evaluated its performance (interrater agreement, intermethod agreement, accuracy, and review time) compared to manual chart review for 4 conditions using randomized 2-period, 2-sequence crossover design.

**Results:**

Case ascertainment with KEEPER was twice as fast compared to manual chart review. 88.1% of the patients were classified concordantly using charts and KEEPER, but agreement varied depending on the condition. Missing data and differences in interpretation accounted for most of the discrepancies. Pairs of clinicians agreed in case ascertainment in 91.2% of the cases when using KEEPER compared to 76.3% when using charts. Patient classification aligned with the gold standard in 88.1% and 86.9% of the cases respectively.

**Conclusion:**

Structured data can be used for efficient and interpretable phenotype evaluation if they are limited to relevant subset and organized according to the clinical reasoning principles. A system that implements these principles can achieve noninferior performance compared to chart review at a fraction of time.

## Introduction

The validity of results of observational studies highly depends on the accuracy of phenotyping algorithms used to identify patients of interest.^1^ Phenotyping can be divided into 2 activities: phenotype development and evaluation. There have been numerous advances in development but the current gold standard for evaluation—chart review—remains time- and labor-consuming, is variable, and requires access to charts, which is not possible on all data sources.

Because chart review is time-consuming, the researchers typically review a small subset of patients identified by the algorithm.^2^^,^^3^ Some studies examine a larger sample size but take months and require significant resources,^4–7^ which is not scalable for more than 1 condition at a time. As only a small sample is typically reviewed, results can suffer from selection bias.^8^ It can be mitigated by condition-specific phenotype-guided chart sampling strategies,^9^^,^^10^ but their generalizability remains limited.

The chart review process can be highly variable due to differences in training, high volume of information in health records, and chart sparsity.^11–13^ If patients are observed in the system regularly, the information volume grows quickly with conflicting information found in different places in the chart.^14^ On the other hand, most of the content in charts is highly redundant and useful information can be buried under duplicated notes.^15^ A growing body of literature uses various natural language processing techniques to extract diagnostic information and reduce volume of information.^16–21^ While these models show high flexibility and adaptability, they tend to be disease-specific, which limits their scalability.

Chart review often requires acquiring additional access to identified unstructured data, which may not be feasible for some researchers or institutions. It is not possible on the data sources with no charts such as administrative claims. While the latter offer more comprehensive patient capture as insurance tracks patients across all institutions, the inference from claims can be perceived as inferior to electronic health record (EHR) because traditional phenotype evaluation is not performed. One potential solution is using linked EHR-claims or registry-claims data sources where the former can act as a gold standard,^22^ but this type of validation is only available in a rather small number of institutions that have linked data sources. Alternatively, researchers used predictive models to derive phenotype performance metrics.^23^ While very promising, such approaches may lack interpretability and transparency.^24^

Due to these limitations, researchers who do not have the time, resources, or infrastructure to conduct chart review either (1) do not evaluate their phenotypes or (2) use phenotype definitions previously described in the literature and rely on previously estimated performance.^25^ Nevertheless, good performance on 1 data source does not guarantee portability to other data sources,^26^^,^^27^ across study periods or populations. Even though further assessment is needed to apply borrowed phenotypes in new settings, it is rarely done.^28^

Therefore, there is a large need for an approach to phenotype evaluation that is scalable and interpretable and can be performed on data sources that lack charts. We propose that structured data (such as ICD10CM codes) can be used to effectively ascertain patient status for phenotype evaluation. We hypothesize that 3 principles are crucial in this process: (1) organization of the data in the way that mimics a typical clinical diagnostic process, (2) presentation of only relevant information as opposed to the whole volume of patient-structured data, and (3) standardization of information extraction and representation. We use these principles to design and evaluate a scalable and interpretable chart review alternative: Knowledge-Enhanced Electronic Profile Review (KEEPER) system.

## Methods

The methods section is organized around (1) the design and development of KEEPER (its principles and application to structured data) and (2) its evaluation (comparison of its performance to manual chart review using 4 conditions of interest as an example).

### KEEPER design and development

We designed KEEPER based on 3 principles: adherence to clinical reasoning, standardization, and dimensionality reduction.

#### Adherence to clinical reasoning

First, we were looking for an interpretable phenotype evaluation solution. KEEPER structure mimics steps of diagnostic clinical reasoning when applied to patient-structured data within the context of the phenotype being evaluated. KEEPER uses the following elements of diagnostic reasoning to organize structured data: clinical presentation (complaints, signs, symptoms, and physical examination), history (disease history, comorbidities, risk factors, and exposures), preliminary diagnosis, subsequent diagnostic procedures, diagnoses, treatment, follow-up care, and complications.

#### Standardization

Second, we needed a scalable solution. In KEEPER, both data extraction and representation are standardized so that it can be used across different data sources and conditions without modification. Standardized extraction is supported by a common data model. In our case, it is Observational Health Data Sciences and Informatics (OHDSI) Observational Medical Outcomes Partnership (OMOP) Common Data Model (CDM), which harmonizes both the structure of the data (common tables and conventions for populating them) and its content through use of common reference system—OHDSI Standardized Vocabularies. Standardized representation is based on the conceptual elements described above.^29^ As the steps of clinical reasoning are universal for any condition,^30^ the structure of data representation is unified and, as a result, disease-agnostic.

#### Dimensionality reduction

Third, a solution had to be efficient (less time-consuming). As the patient data are reviewed for the purpose of phenotype evaluation, we only extract the information clinically relevant to a given phenotype. Reducing data volume potentially facilitates review and makes it faster. We hypothesize that the structured data provides sufficient information to ascertain patient status even despite previously observed data loss.^31^

Following these principles, KEEPER uses structured data standardized to a common format to extract data elements that correspond to the typical steps clinicians follow when diagnosing a patient, selects only information relevant to a given phenotype, and outputs data in a standardized format. Specific data elements depend on a condition of interest but correspond to common disease-agnostic conceptual elements or ideas (Table 1).

**Table 1.** Conceptual elements and corresponding data elements in KEEPER, example for acute appendicitis phenotype

Conceptual element	Conceptual element in the context of the disease of interest	Data element
Clinical presentation	Presence of relevant [known to be associated with the outcome] symptoms on the encounter (index date, day 0) and absence of competing symptoms	Condition codes [day 0] *Nausea, vomiting, epigastric pain*
Clinical plausibility	Appropriate demographics	Age, gender, race, and ethnicity [day 0] *Male, 37 yo, race = White*
	Presence of relevant symptoms, diagnoses, or treatment prior to the index date, especially recurring	Condition, drug, and observation codes [before day 0] *None expected*
	Presence of relevant co-morbidities and (or) predisposing risk factors	Condition and observation codes [before day 0] *Family history of acute appendicitis*
	Absence of competing diagnoses after the index date, especially if followed by treatment	Condition, procedure, measurement, and drug codes [after day 0] *Absence of diagnosis of Crohn’s disease, cancer of colon, etc.*
Diagnostic procedures	Presence of diagnostic procedures, laboratory tests, clinical consults with other specialties, and transfer to specific care sites around the index date	Procedure codes [before and after day 0] *X-ray of abdomen*
		Measurement codes and values [before and after day 0] *Leukocytosis*
		Provider and location [before and after day 0] *Emergency room, operating room*
Treatment procedures and medications	Presence of relevant instrumental and surgical procedures performed on or after the index date	Procedure codes [after day 0] *Appendectomy*
	Presence of relevant medications prescribed or administered on or after the index date	Drug codes [after day 0] *Oral antibiotic therapy*
Follow-up care and complications	Presence of relevant follow-up visits	Provider and location [after day 0] *None expected*
	Presence of relevant complications after the index date	Condition codes [after day 0] *Postsurgical complications*

The first conceptual element is clinical presentation, which consists of patient’s symptoms, signs, and complaints on the day they seek care (day 0 or index date). In clinical practice, a physician (or healthcare team) collects current complaints, past personal and family history, assesses vital signs, performs physical examination, and, based on the totality of information, makes a preliminary diagnosis.

For example, in the context of acute appendicitis phenotype, Patient X with suspected acute appendicitis (in textbook scenario) presents to the emergency room complaining of epigastric pain migrating to right lower quadrant, nausea and vomiting. Physical exam reveals fever, localized tenderness in the right lower quadrant and positive Rovsing's sign.^32^

On the data level, it translates into condition codes for corresponding signs and symptoms (such as ICD-10(CM) R11.0 “Nausea”), measurement codes for vital signs (such as high body temperature), or condition codes for acute appendicitis. Observing these data elements increases one’s confidence in the diagnosis and observing symptoms typical for other conditions (such as intermittent severe pain that waxes and wanes in renal colic) or competing diagnoses (diverticulitis or renal colic) decreases one’s confidence.

The second conceptual element is clinical plausibility, which includes specific demographics if a condition is known to be prevalent in a given group, history of disease, and predisposing factors. Within the context of acute appendicitis phenotype, Patient X is more likely to be young^33^ and less likely to have prior recurrent abdominal symptoms or have been diagnosed with Crohn’s disease or endometriosis. If a condition of interest was chronic or had known risk factors, one would expect to observe prior episodes of care or relevant comorbidities. On contrary, observing a competing diagnosis recorded after the encounter (such as Crohn’s disease), especially followed by the subsequent treatment would decrease one’s confidence in the diagnosis.

The next conceptual element encompasses diagnostic procedures and laboratory tests. In our clinical scenario, Patient X is sent for blood work and diagnostic imaging of the abdomen (ultrasound or computer tomography). Diagnostic findings include leukocytosis and radiographic signs of appendicitis (enlarged appendix with wall thickening or perforated appendicitis). From the data perspective, observing these diagnostic procedures (such as CPT4 74160 “Computed tomography, abdomen; with contrast material(s)”) along with corresponding laboratory values (such as LOINC-coded measurements and their results) would increase one’s confidence in the diagnosis.

Treatment procedures and medications are approached in the same way. Subsequent treatment can include a short course of antibiotics (eg, piperacillin-tazobactam or cephalosporins in combination with metronidazole), appendectomy within a day or interval appendectomy. In our scenario, Patient X undergoes laparoscopic appendectomy and pathologic examination of the appendix reveals gangrenous appendicitis. Since the final pathologic diagnosis is consistent with acute appendicitis, the clinical case can be concluded. As pathology and operative reports are oftentimes not available in the structured data, observing relevant treatment and complications of appendicitis (such as CPT4 44950 “Appendectomy”) along with absence of competing treatment (such as colectomy or gastrotomy) would conclude the case in the structured data.

These conceptual elements along with their data representation form a basis of KEEPER. It takes a cohort of patients identified based on the phenotype definition that one wants to evaluate along with the specific codes for data elements (defined by a user) and outputs KEEPER profiles. Table 2 shows the examples of such for 3 patients with suspected acute appendicitis. The records do not reflect real patient data but are constructed based on the data from the cases we reviewed. The first patient in Table 2 (green) is 46-year-old male, admitted with abdominal pain, enlarged liver and leukocytosis. Clinical presentation is consistent with acute appendicitis or umbilical hernia, so the patient is referred to computer tomography of abdomen and is treated with a short course of antibiotics. Subsequently, the patient is diagnosed with acute gangrenous appendicitis and undergoes appendectomy. Presence of relevant symptoms, diagnostic and treatment procedures and absence of competing diagnoses after the index date is highly suggestive of acute appendicitis.

**Table 2.** Examples of KEEPER profiles for 3 patients with suspected acute appendicitis: likely a case (green), likely a control (red) and ambiguous (blue)

Demographics and details about the visit	Presentation	Prior conditions, symptoms and treatment	Diagnostic procedures	Laboratory tests	Competing diagnoses	Treatment procedures and medications	Complications
Male, 46 yo; visit: emergency room followed by hospitalization (3 days)	Abdominal pain; acute appendicitis; large liver; umbilical hernia without obstruction and without gangrene	Abdominal pain (day −71); abdominal pain (day −1)	Computed tomography, abdomen and pelvis; with contrast material(s (day 0);	Leukocytes (abnormal, high, day 1); neutrophils (normal, day 1); neutrophils/100 leukocytes (abnormal, high, day 1)		Appendectomy (day 25); metronidazole (3 days)	Acute gangrenous appendicitis (day 25); acquired absence of organ (day 25)
Female, 17 yo; visit: hospitalization (7 days)	Abdominal pain; appendicitis; diverticulitis of colon; fever	Diverticulitis of colon (day −182)	Computed tomography, abdomen; with contrast material(s); computed tomography, pelvis; with contrast material(s) (day 5)	Leukocytes (abnormal, high, day 0/1/2/5); leukocytes (normal, day 3/4/6/7); neutrophils/100 leukocytes (normal, day 0/6); neutrophils/100 leukocytes (abnormal, high, day 1-5)	Diverticulitis of colon (day 20)	piperacillin and tazobactam (5 days)	
Male, 70 yo; visit: emergency room followed by hospitalization (2 days)	Acute appendicitis; Barrett's esophagus; esophagitis; gastrointestinal hemorrhage; hematemesis	Abdominal pain (day −816); esophagitis (day −180)	Esophagogastroduodenoscopy, flexible, transoral; diagnostic, including collection of specimen(s) by brushing or washing, when performed (day 0)	Leukocytes (abnormal, high, day −1 and 0); leukocytes (normal, day 1); neutrophils (normal, day −1); neutrophils/100 leukocytes (normal, day −1)	Diaphragmatic hernia; Barrett's esophagus; hematemesis; eosinophilic esophagitis; gastrointestinal hemorrhage	Pantoprazole (62 days); famotidine (2 days); ondansetron (1 day)	

On the contrary, the last patient in Table 2 (in red) is likely a control. Seventy-year-old man presented to the emergency department with symptoms suggestive of an acute abdominal problem (acute appendicitis, Barrett's esophagus, and esophagitis). Given the presence of hematemesis (a serious potentially life-threatening acute event with clear unambiguous presentation), we can suspect that hematemesis was the main complaint and acute appendicitis was a rule-out diagnosis. Subsequent diagnostic procedures (presence of esophagogastroduodenoscopy for hematemesis and absence of computer tomography for appendicitis) and treatment (acid-reducing drugs) likely confirm that this patient did not have acute appendicitis.

The other patient has the elements suggestive of appendicitis (laboratory findings and appropriate treatments) but also has the elements indicative of another condition (history of diverticulitis and subsequent diagnosis of diverticulitis), so the choice regarding the status of such patient is left to the reviewer’s discretion.

Examples for other conditions are provided in Table S1.

### KEEPER evaluation

**Figure 1.**
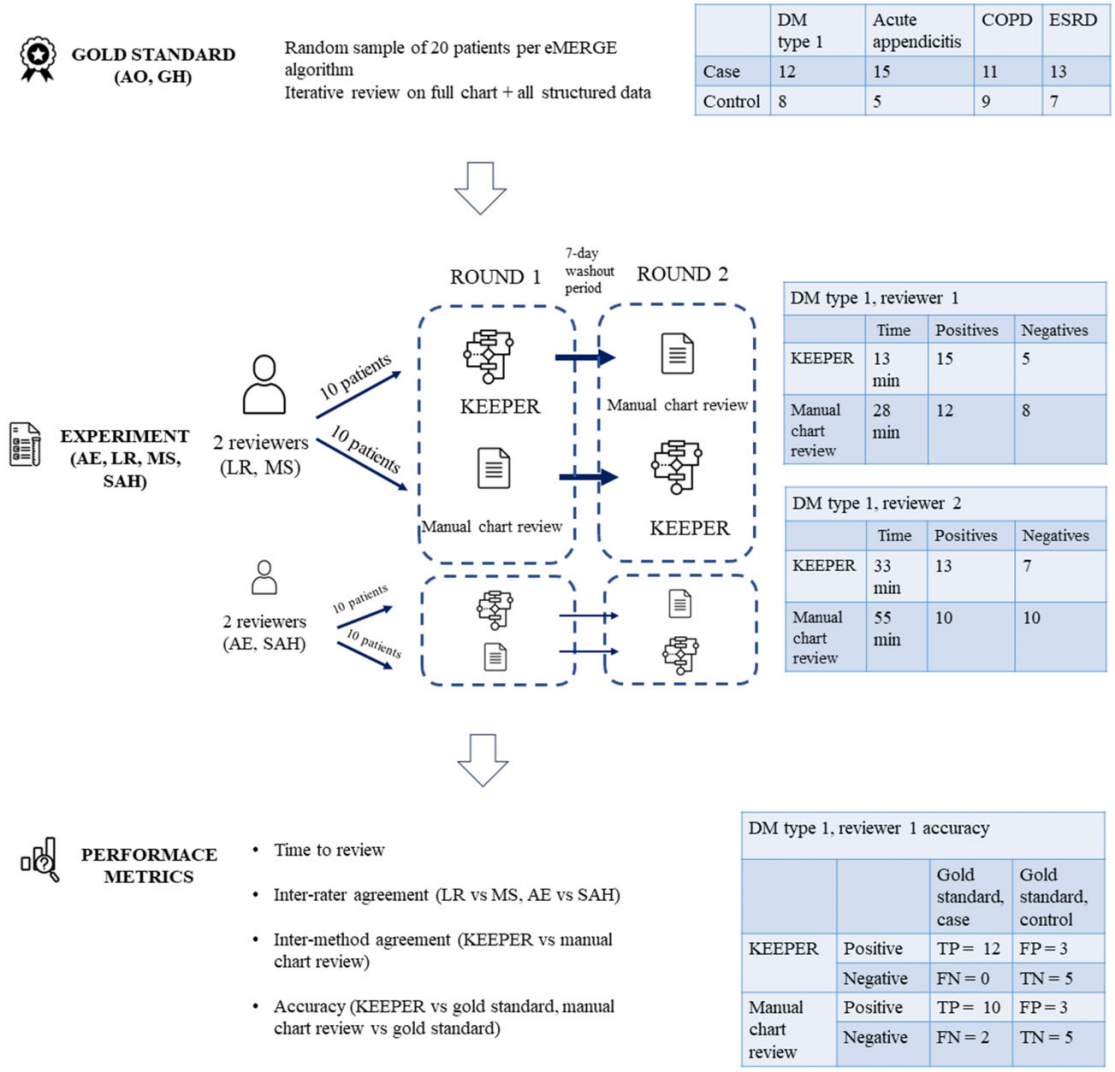
Overview of the proof-of-concept experimental design for comparing KEEPER and manual chart review for phenotype evaluation.

As a proof-of-concept study, we ran KEEPER for 4 phenotypes and conducted a randomized standardized experiment comparing the performance of KEEPER profiles and manual chart review. We selected conditions that represent chronic and acute conditions, rare and prevalent, those that are usually managed in inpatient and outpatient settings: acute appendicitis, diabetes mellitus type I (DM type I), chronic obstructive pulmonary disorder (COPD), and end-stage renal disease (ESRD). Figure 1 shows the overview of study design.

#### Data source

We used Columbia University Irving Medical (CUIMC) EHRs translated to OMOP CDM.^29^ The database has electronic health records and records from administrative and ancillary systems for more than 6 million patients and currently holds information about the person (demographics), visits (inpatient and outpatient), conditions (billing diagnoses and problem lists), drugs (outpatient prescriptions and inpatient orders and administrations), devices, measurements (laboratory tests and vital signs), and other observations (symptoms). Some of the elements not captured in our OMOP structured data include results of imaging studies, bacterial culture tests, or content of free-text notes.

#### Gold standard

For each disease, we executed eMERGE algorithms that were previously developed and validated on CUIMC data^34–38^ and selected a random subset of 20 patients for each condition (all patients were cases according to the algorithms). Two authors (A.O. and G.H.) independently performed chart review on full patient medical records and all available structured data and classified each patient as case or control. Results were discussed and iterative chart review continued until all disagreements were resolved. Figure 1 shows the distribution of cases and controls in gold standard for each disease.

#### Test set

AO created KEEPER profiles for each patient from the gold standard. Data extraction for KEEPER was performed uniformly for all patients prior to the experiment and KEEPER profiles were not modified based on the results of chart review for gold standard.

Input for KEEPER (set of codes for each data element) was constructed in a semiautomated fashion. Demographic characteristics and recorded symptoms, signs, and diagnoses on day 0 were extracted from OMOP CDM *person* and *condition_occurrence* tables without any modification. Relevant comorbidities, disease history (recorded any time before the index date), differential diagnoses, and complications (any time after the index date) were extracted from *condition_occurrence* table, where selection was guided by the SNOMED-CT hierarchy and refined iteratively based on the distribution of the concepts in CUIMC EHR data. For example, for acute appendicitis we extracted all descendants of SNOMED-CT “Disorder of abdomen,” “Disorder of pelvis,” and “Disorder of the genitourinary system.” Risk factors such as smoking for COPD were extracted from *observation* table. Relevant drugs (recorded any time on or after the index date) were extracted using the joint ATC-RxNorm hierarchy using grouping terms in ATC (eg, all descendants of ATC “Antiinfectives for systemic use” and “Alimentary tract and metabolism” for acute appendicitis) and presented at the ingredient level with days supply.

Procedures and measurements (laboratory tests and vitals recorded before, on, and after the index date) were defined in groups based on clinical expertise. The codes can be found on GitHub.^39^

Input codes were fed into KEEPER to produce KEEPER profiles for all patients in test set (structured similarly to Table 2) and distributed to reviewers as described below.

#### Experiment

We followed 2-period, 2-sequence crossover design, where 2-period refers to 2 rounds and 2-sequence refers to the order of studied methods.^40^ Four clinicians (L.R.R., M.S., S.A.H., A.E.) independently reviewed KEEPER profiles and manually reviewed patient charts, 2 for acute appendicitis and DM type 1 phenotypes, and the other 2 for COPD and ESRD.

For each condition, we randomly split 20 patients into 2 groups of ten, so that during the first round a reviewer ascertained KEEPER profiles of patients 1-10 and manually reviewed charts of patients 11-20 and during the second round—KEEPER profiles of patients 11-20 and charts of patients 1-10. There was a minimum of a 7-day wash-out period between rounds. Patients were assigned different identifiers to prevent carryover effect.

AO and GH did not participate in review for KEEPER evaluation and did not modify the gold standard or KEEPER profiles throughout the experiment.

#### Metrics

First, we calculated the proportion of patients classified concordantly by manual chart review and KEEPER profile review (intermethod agreement). We used Cohen’s kappa (chance-corrected agreement) for each condition as well as the overall agreement.

Second, we measured interrater agreement between 2 study reviewers to assess if consistency of case ascertainment among reviewers is improved by using KEEPER profiles. As we used fully crossed design, Fleiss’s kappa was chosen as the metric for the overall agreement and Cohen’s kappa for pairwise comparison.^41^ The Cochran-Mantel-Haenszel test was used to compare methods across different conditions followed by Fisher exact test for pairwise comparisons.^42^

Third, we compared the accuracy of manual chart review and KEEPER review compared to the gold standard, where the accuracy was calculated as the proportion of the labels (cases and controls or positives and negatives) that agree with the gold standard. Proportions were compared using the Cochran-Mantel-Haenszel test.

Finally, we compared the time needed to review KEEPER profiles and charts using the Student’s t-test and performed qualitative analysis of the discrepancies in case ascertainment.

## Results

### Efficiency

The use of KEEPER reduced the time needed for review in more than half in both rounds. On average, chart review for 20 patients took 67 min (SD = 43) and KEEPER profile review took 30 min (SD = 14, *P* value .04).

Review time did not differ significantly in the first and the second round for both charts (mean [SD] = 72.8 min [45.6] in the first round and 61.0 min [47.6] in the second round) and KEEPER profiles (32.3 min [14.0] and 28.3 min [16.3], respectively).

### Agreement

We observed substantial agreement between the results of manual chart review and KEEPER profiles (Table 3). Overall, 88.1% of the cases were ascertained similarly using charts and KEEPER, which corresponded to Cohen’s kappa of 0.71 (95% CI 0.59-0.83).

**Table 3.** Comparison of manual chart review and KEEPER profiles: intermethod agreement, interrater agreement, and accuracy

	Intermethod agreement		Interrater agreement				Accuracy	
	Cases, *n* (%)	Kappa (95% CI)	Chart, *n* (%)	Kappa (95% CI)	KEEPER, *n* (%)	Kappa (95% CI)	Chart, *n* (%)	KEEPER, *n* (%)
DM type I	32 (80.0)	0.58 (0.34-0.82)	14 (70.0)	0.40 (<0.1 to 0.78)	18 (90.0)	0.77 (0.47 to 1.00)	34 (85.0)	35 (87.5)
Acute appendicitis	38 (95.0)	0.87 (0.69-1.00)	19 (95.0)	0.86 (0.56 to 1.00)	19 (95.0)	0.88 (0.64 to 1.00)	39 (97.5)	39 (97.5)
COPD	34 (85.0)	0.67 (0.44-0.90)	16 (80.0)	0.60 (0.28 to 0.92)	20 (100.0)^a^	1.00 (1.00 to 1.00)	34 (85.0)	32 (80.0)
ESRD	37 (92.5)	0.78 (0.54-1.00)	12 (60.0)	−0.1 (−0.3 to 0.1)	15 (75.0)	0.34 (−0.01 to 0.72	32 (80.0)	35 (87.5)
Overall	141 (88.1)	0.71 (0.59-0.83)	61 (76.3)	0.45 (0.23 to 0.67)^b^	73 (91.2)^a^	0.74 (0.52 to 0.96)^b^	139 (86.9)	141 (88.1)

Kappa ≤0 indicates no agreement; 0.01-0.20 indicates none to slight; 0.21-0.40 indicates fair; 0.41-0.60 indicates moderate; 0.61-0.80 indicates substantial; and 0.81-1.00 indicates almost perfect agreement.

a Significant difference between 2 methods based on Cochran-Mantel-Haenszel test and Fisher exact test (alpha = 0.05).

b Fleiss’s kappa to account for 2 pairs of reviewers; other kappas are Cohen’s kappa.

Overall, KEEPER allowed a researcher to arrive at the same conclusions regarding patient status as manual chart review in 80% of the cases. Still, we observed heterogeneity in agreement across conditions. The lowest agreement was observed for diabetes mellitus type I (moderate agreement) and the highest—for acute appendicitis (almost perfect agreement).

When comparing interrater agreement (agreement in case ascertainment between 2 reviewers), we observed that KEEPER enabled more consistent review. Reviewers arrived at the same conclusions regarding the patients’ status in 91.2% of the cases when using KEEPER compared to 76.3% in manual chart review. This trend was observed for diabetes mellitus type I, end-stage renal disorder, and chronic obstructive pulmonary disorder.

### Accuracy

Finally, the accuracy of KEEPER was noninferior to manual chart review. Overall, in 88.1% and 86.9% of cases, respectively, case ascertainment aligned with the gold standard. In all conditions, accuracy of KEEPER was at least 80%. Table 4 provides the analysis of sources of discrepancies in case ascertainment.

**Table 4.** Categories of sources of discrepancy in case ascertainment when comparing (1) chart review and gold standard, (2) KEEPER profiles and gold standard, and (3) chart and KEEPER (indirect comparison)

Chart vs Gold standard	KEEPER vs Gold standard	Chart vs KEEPER
(1) High chart volume - Study reviewers missed records of cancer of ileum appendix (condition of exclusion) in patient records and classified acute appendicitis control as case. - Study reviewers missed records of diabetes type II (condition of exclusion) in early patient records and classified DM type I control as case. - Study reviewers missed records of asthma (condition of exclusion) and supporting treatment (montelukast) in early patient records and classified COPD control as case. (2) Conflicting information - Measurement of old pulmonary function in charts suggested asthma (condition of exclusion) while diagnoses codes suggested COPD, COPD control was classified as case.	(1) Missing data in KEEPER - Reviewers used short-term antibiotic treatment along with normal white cell count to classify the case as control. As pathology report was not available, they suspected that it would be negative for appendicitis and classified appendicitis case as control. - Early notes about asthma (condition of exclusion) were missing in KEEPER, reviewer classified COPD control as case. (2) Interpretation - Study reviewers did not infer secondary diabetes (condition of exclusion) from previous alcohol-induced pancreatitis and classified DM type I control as case. - Study reviewers did not infer steroid-induced diabetes (condition of exclusion) from previous prolonged steroid exposure and short insulin treatment and classified DM type I control as case.	(1) Interpretation - Chart had a narrative about obstruction caused by cancer (exclusion for COPD), which was not available in KEEPER. Objective tests were not available in KEEPER or charts. (2) High chart volume - KEEPER presented cancer diagnosis as a relevant competing diagnosis for acute appendicitis while finding the diagnosis in chart required additional exploration. (3) Missing data in KEEPER - Pathology report with signs of acute appendicitis was available in charts but not in KEEPER. - Indicators of specialty and location of visit were missing in KEEPER, which did not allow study reviewers to meaningfully assess discrepancies between specialty diagnoses and GP diagnoses for DM type I. - Pancreatectomy due to necrotizing alcohol-induced pancreatitis (exclusion for DM type I) was available in charts but not in KEEPER.

## Discussion

This study aimed to assess if structured data organized in a standardized fashion according to the clinical reasoning principles can support efficient, interpretable, and scalable phenotype evaluation. It has long been posited that crucial information about the patient state, diagnoses, and symptoms is most fully and accurately recorded in unstructured free-text notes and that only the notes can serve as the gold standard in phenotype evaluation. Indeed, unstructured data offers great opportunity for expression, allowing clinicians to both interpret other providers’ narratives and create their own.^24^ As a result, there have been multiple disease-specific endeavors in natural language processing aiming at improving phenotype development and evaluation by capturing richness of free text.^20^, ^43–45^

Yet, we need a scalable solution. KEEPER represents only relevant data in a structured way, which decreases time to review, and which supports previous findings on benefits of standardized practices for case ascertainment.^46^^,^^47^ As a result, researchers can review more patients with KEEPER, thus enabling more reliable estimation in clinical studies. It can be especially useful in safety research where rare outcomes require larger sample sizes.^48^

One of the factors that consistently facilitated faster case ascertainment was dimensionality reduction. Patient-structured data can contain hundreds and thousands of events, codes, and values, which decreases the efficiency of review and increases the likelihood of missing important information. Similarly, high volume of information and contradicting information in charts were a source of disagreement among reviewers (Table 3). For example, COPD has to be differentiated from asthma, which requires the assessment of history of disease, pulmonary tests and previous drug exposures. In our patient sample, some patients with bronchial obstruction did not have a history of asthma in the recent notes but previous notes (sometimes going back 10 years and more) had a diagnosis of asthma, montelukast (a drug almost exclusively used for mild and intermittent asthma) or bronchodilator use, which undermined the reliability of the later diagnosis of COPD. Finding this information required scrutinizing tens of clinical notes, which lengthened reviews and decreased accuracy.

Another source of disagreement among reviewers in manual chart review could be differences in clinical training and expertise and different approaches to chart review. One scenario used by study reviewers involved reviewing patient data around the day 0 first, moving sequentially along the longitudinal patient record. Another scenario involved starting at the data elements that carried the most accurate perceived information (such as pathology reports for acute appendicitis or specialty notes associated with laboratory values for the other conditions) and then retrospectively reconstructing the case. Standardization of data representation in KEEPER partially mitigated this issue leading to higher interrater reliability.

We proposed that standardizing the input and output of KEEPER facilitates scalability of chart review as the former has a potential to perform similarly across a broad range of conditions. While examining this hypothesis on all possible conditions is not feasible, we selected a mix of chronic and acute, inpatient and outpatient conditions to cover a variety of conditions. On the one hand, we observed consistent improvement in interrater reliability across all conditions, which strengthened our assumption that KEEPER can be seen as a disease-agnostic solution. On the other hand, heterogeneity of intermethod agreement and accuracy of different conditions points at the need for more research on factors influencing inference from structured data and potentially limits applicability of KEEPER. In COPD, the factors that contributed to lower accuracy compared to other conditions included inability to (1) easily interpret the results of pulmonary function tests to distinguish COPD from asthma or chronic bronchitis and (2) ascertain the cases when no results of pulmonary function tests were available. Similar challenges were encountered in manual chart review, especially if the results of pulmonary function tests were contradictory or inconclusive.

KEEPER seems to be efficient if the structured data contain the necessary elements for valid inference and therefore its performance may depend on comprehensiveness of data capture and specifics of patient population in a given data source. Data are likely to be sufficient to infer prevalent conditions and conditions requiring drug therapy or operative procedures.^49–51^ On contrary, it is commonly acknowledged that asymptomatic conditions and some comorbidities are underrepresented in structured data.^52^ Similarly, structured data and billing codes are not likely to capture conditions associated with privacy concerns.^53^ It is not clear to what extent the performance observed in this study can be replicated on claims data sources for those conditions whose diagnosis is heavily measurement-based. In our example, sensitivity of KEEPER may be low when attempting to ascertain patients with COPD or ESRD on claims data sources as there are patients who do not receive specific treatment and, therefore, can be misclassified as controls.

In the future, we envision KEEPER as a user interface integrated in a broader stack of OHDSI tools, which will enable seamless integration of phenotype development, cohort execution, cohort diagnostic, and phenotype evaluation.^23^, ^54–56^ For this proof of concept, we used a semiautomated (data-driven, ontology-guided code selection with subsequent review) approach to extract relevant information. In the future, for KEEPER to be scalable, relevant information must be extracted in an automated disease-agnostic fashion. There are many works on identifying similar concepts, including lexical, ontological, and data-driven approaches^57–59^ that can be leveraged to accomplish this task. Given complexity of the task, an appropriate method should be able to identify relevant but not necessarily semantically similar concept, concepts from different domains (such as laboratory tests relevant to a given disease), and clinically meaningful concept pairs (such as diagnosis-differential diagnosis pairs^60^)

### Limitations

This study has a number of limitations. First, the study was conducted on rich data from a tertiary healthcare center. Therefore, our findings may not be generalizable to the institutions with higher expected information loss from charts to structured records. Similarly, the results may not be generalizable to the data sources that have different data elements captured (eg, to those that do not have pharmacy claims or drug prescription records).

Second, we conducted the experiment for 4 conditions and while these conditions represent a spectrum of disorders requiring different levels and settings of care, the results may not be generalizable to other conditions. As we observed heterogeneity in performance for different conditions, it is likely that observed performance will not be applicable to the conditions that are associated with low healthcare utilization, asymptomatic conditions or underreported conditions.

Third, while we wanted to enable interpretable phenotype evaluation on the data sources that lack charts, KEEPER performance on such was not explicitly examined.

Fourth, this study did not use fully specified chart review protocols. Comparative performance of KEEPER and manual chart review could be different if reviewers are provided with fully specified protocols for chart review. Similarly, the reviewers had different training and expertise, which potentially influenced study results.

Fifth, only patients determined to have a condition by a corresponding eMERGE algorithm (“positive”) were selected for this study. Since patients classified as not having a condition (“negative”) were not sampled for this study, performance of KEEPER profiles for such patients was not estimated.

Next, our gold standard was constructed based on iterative review of full charts and all structured data. As opposed to individual manual chart review in the experiment, A.O. and G.H. extensively discussed each case until disagreements were resolved. Nevertheless, chart review was used for both gold standard and experiment, so accuracy estimates should be treated with caution.

Finally, while KEEPER enables faster review compared to manual chart review, there was an additional cost of developing input for KEEPER. Future work includes developing data-driven approaches to code selection to decrease this cost.

## Conclusions

Phenotype evaluation remains the bottleneck of observational research as the current gold standard—chart review—is interpretable and trusted but expensive and time-consuming, and requires access to charts. In this study, we evaluated the ability of structured data to support effective case ascertainment. We used the principles of clinical reasoning, standardization, and dimensionality reduction to build a Knowledge-Enhanced Electronic Profile Review system or KEEPER. We demonstrated that structured data can support valid inference about patient state if organized and presented according to these principles. KEEPER showed noninferior performance compared to chart review at a fraction of time. It, therefore, can enable more scalable phenotype evaluation on electronic health records and sources that lack charts (such as administrative claims data sources).

## Ethical approval

We obtained an approval to conduct this research from the Columbia University Medical Center institutional review board (IRB-AAAS6414).

## Supplementary Material

ocad202_Supplementary_Data

## Data Availability

The data used in this study is protected health information and cannot be made publicly available.
